# Medical Coaching for Psychiatrists

**DOI:** 10.1192/bjo.2024.287

**Published:** 2024-08-01

**Authors:** Jenny Forge, Peter L Cornwall

**Affiliations:** Tees, Esk and Wear Valleys NHS Trust, Middlesbrough, United Kingdom

## Abstract

**Aims:**

Promoting recruitment and retention in psychiatry is one of the core objectives for the Royal College of Psychiatrists and coaching initiatives are recognised as a means of improving retention. We developed a programme of medical coaching, available to all career-grade doctors in an NHS Trust in Northern England to support professional development. This overview describes the results of the first 4 years of the programme.

**Methods:**

The setting was a large NHS Trust covering County Durham, Teesside and North Yorkshire employing around 150 consultants and 60 SAS psychiatrists (mean age = 49 years, 51% female). Coaching was promoted to all these doctors through the feedback form sent following their annual appraisal meeting. This coaching was later also made available to locally employed doctors and core and higher specialist trainees working temporarily in the Trust. The intervention was initially provided as a single session coaching event delivered by a consultant psychiatrist trained in medical coaching, and the programme evolved following requests from doctors. It was stated explicitly that the purpose was professional development, not an attempt to retain doctors considering their future. The outcome was measured using a post-coaching questionnaire.

**Results:**

Data was collected from coaching delivered from May 2019 to January 2024. 145 doctors (84 consultants, 23 SAS doctors, 6 Trust doctors, 26 training-grade doctors, 6 grade not-stated) took up the coaching offer. 524 sessions were provided in all. The mean (SD) number of sessions was 3.8 (3.7), for consultants 3.5 (3.9) and for SAS doctors 4.8 (4.4). 48 doctors accessed a single coaching session. 56% of the career-grade doctors receiving coaching were female. Data was collected from 127 post-coaching questionnaires with 116 strongly agreeing and 11 agreeing with the statement that the coaching provided was useful and many reporting a positive impact on well-being.

**Conclusion:**

Findings show that the programme was popular with the medical workforce, with about half of career grade psychiatrists taking up the offer. It evolved following requests to both provide follow-up sessions and to extend the offer to trust doctors and trainees. The sessions were highly valued by the doctors with reported benefits to their well-being, but we cannot measure the impact on retention. The programme is valued by the Trust with an intention to make the programme sustainable into the long term and it now forms part of the Trust's medical workforce charter.

